# The Secretion Process of Liquid Silk with Nanopillar Structures from *Stenopsyche marmorata* (Trichoptera: Stenopsychidae)

**DOI:** 10.1038/srep09237

**Published:** 2015-03-18

**Authors:** Tomohiro Hatano, Takayuki Nagashima

**Affiliations:** 1 Tokyo University of Agriculture, Atsugi, Kanagawa 243-0034, Japan

## Abstract

*Stenopsyche marmorata* larvae spin underwater adhesive silk for constructing nests and capture nets. The silk can be divided into fiber and adhesive regions, according to their function. The silk fiber region has a two-layer structure: a core layer situated at the center of the fiber and *S. marmorata* fibroin, the major component of the silk. In the anterior part of the anterior silk gland, the morphological characteristics suggest that the silk insolubilization leading to fibrillation occurs by luminal pH neutralization. The adhesive region is composed of three layers: the outermost (OM), B, and C layers. On the B layer, coated with the OM layer, numerous nano-order pillar structures (nanopillar structures) are located at regular intervals. A nanopillar structure is approximately 40 nm in diameter and 125 nm in length. The precursor materials of the nanopillar structure are electron-dense globules of approximately 25 nm in diameter that are located in the A layer of the lumen of the middle silk gland. The precursor globules autonomously connect to one another on the B layer when the liquid silk is transported to the lumen of the bulbous region. The nanopillar structures probably contribute to the strong underwater adhesion of *S. marmorata* silk.

Trichoptera (caddisfly) are one of the largest orders of aquatic insects. Its larvae are widely distributed in various freshwater bodies, and a few species inhabit marine environments. Trichoptera larvae construct retreats and cocoons with sand and gravel or plants using underwater adhesive silk. The silk is also used as capture nets to filter food particles such as plant detritus by most Hydropsychoidea (the superfamily of *S. marmorata*)^1^.

The adhesive mechanism of Trichoptera silk is interesting from the standpoint of developing novel biomimetic adhesives. Trichoptera silk has an ability to adhere to diverse underwater substrates: stones, leaves, pieces of glass and plastic beads, PTFE, and others^2^,^3^, which is permanent adhesion and not reversible stickiness. In studies on barnacle adhesive proteins, the underwater adhesive is an insoluble multiprotein complex, and each component protein of the complex is suggested to have a special function in a multifunctional process of underwater attachment^4^. The insolubility and multifunctionality of the underwater adhesive protein have been obstacles to the detailed analysis of its function and especially to the direct study of the adhesive process^4^,^5^. Since Trichoptera silk also has been suggested to have strong insolubility^6^, investigating the silk composition and the secretion process is instructive to understand the adhesion mechanism and each constituent protein function.

Trichoptera silk is produced by the labial salivary gland and spun from an orifice of the labium, similar to the sister order Lepidoptera^7^,^8^. The domesticated silk moth *Bombyx mori* has been extensively studied with respect to the silk fiber and silk gland. Detailed ultrastructural knowledge of *B*. *mori* silk structure and the silk secretion process has been accumulated. The *B*. *mori* silk gland consists of three regions: the posterior silk gland (PSG), the middle silk gland (MSG), and the anterior silk gland (ASG). Microscopic studies show that PSG secretes fibroin and MSG secretes gelatinous sericin^9^. The fibroin inner core and the outer coating of sericin layers are observed in a cross section of *B*. *mori* cocoon filament. The sericin functions as adhesive material, which glues a pair of fibroin layers and the cocoon to its pupation site^10^. Spider silk functioning as an adhesive secreted from pyriform^11^,^12^ and cylindrical gland^13^ consists of two component compositions similar to *B. mori* silk, which are composed of core fiber and an amorphous coating.

The *B. mori* sericin is divided into inner, middle, and outer sericin layers based on the histological staining properties of the liquid silk in MSG^14^. The histochemical study and transmission electron microscope (TEM) observations suggest that the sericins in the inner, middle, and outer layers are secreted in the posterior, middle, and anterior parts, respectively, of MSG^15^,^16^. The middle part of MSG is further divided into the posterior and anterior sections, of which the former secretes exclusively middle-layer sericin and the latter secretes both middle- and outer-layer sericin^15^. SDS-PAGE analysis has shown that the principal components of sericin are sericin P (150 kDa), sericin M (400 kDa), and sericin A (250 kDa), located largely in the posterior, middle, and anterior part of MSG, respectively^17^; these results are consistent with microscopic studies. In this way, microscopic studies are a useful analysis technique for studying the hierarchical structure and components of multiprotein silk.

Except for *B. mori* and spider there are few studies that have microscopically characterized the relationship between types of silk secretion and ultrastructure of the silk gland. Engster (1967) observed the silk gland of two Limnephilidae caddisflies, *Pycnopsyche guttifer* and *Neophylax concinnus*, by TEM^18^. The silk glands were distinguishable into three anatomical regions: a silk secretion area that occupies most of the silk gland, the anterior conducting tube, and the common conduct area of the anterior end^18^. The liquid silk in the lumen was observed to form two layers: a “core” and a “peripheral” layer corresponding to fibroin and sericin, respectively^18^. *Stenopsyche marmorata* (Stenopsychidae) is a common species of caddisfly in Japan and is suitable as an experimental material because its large population and larval body. In a previous study we showed adhesion interface of *S. marmorata* silk by TEM observation^2^. This report describes the ultrastructure of the liquid silk and silk secretion in the silk gland of *S. marmorata*.

## Results

**Figure 1:**
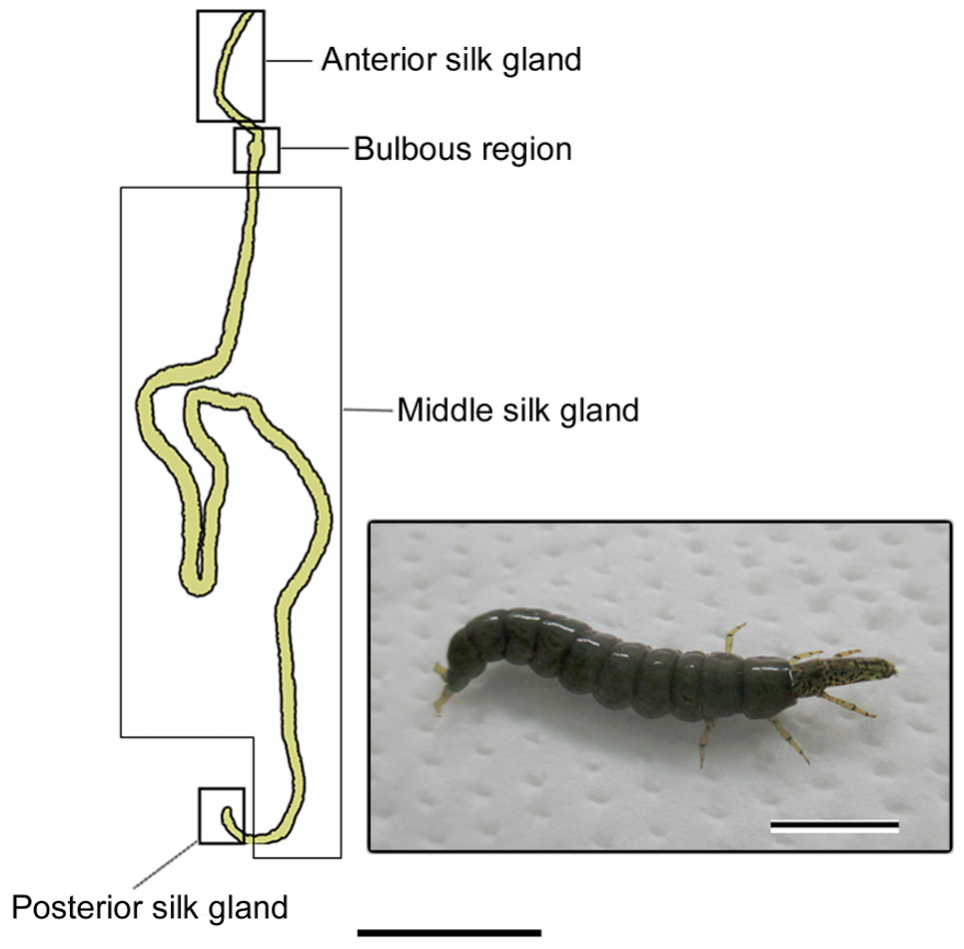
Schematic representation of *Stenopsyche marmorata* silk gland. The silk gland may be divided into four sections according to differences in silk secretion and anatomical features. Scale bar, 0.5 cm. The inset shows the fifth instar larva. Scale bar, 1 cm.

The silk gland could be divided into four divisions on the basis of the types of silk secretion and the histological features: the posterior silk gland, the middle silk gland, the bulbous region, and the anterior silk gland (Fig. 1).

### Posterior silk gland

**Figure 2:**
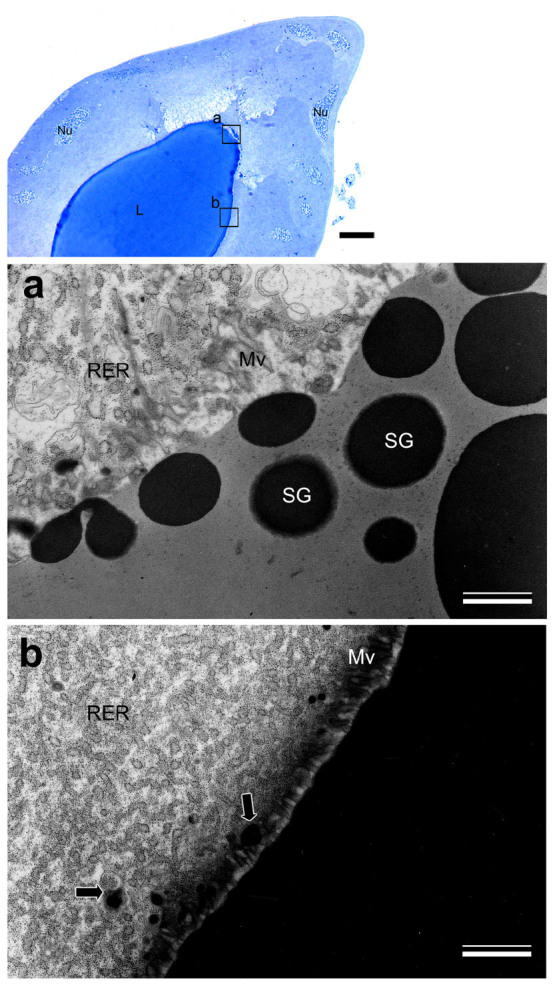
Posterior silk gland of *S. marmorata*. Above is a light micrograph of the longitudinal section. Scale bar, 20 μm. Below are the electron micrographs of the boxed area in the upper figure. (a) Electron-dense secretory globules (SG) of core layer components are seen in the lumen. Scale bar, 1 μm. (b) The lumen is completely filled with core-layer components by accumulation of the secretory globules. Core-layer components are seen in the apical cell (arrow). RER: rough endoplasmic reticulum; L: lumen; Mv: microvilli; Nu: nucleus. Scale bar, 1 μm.

In the posterior silk gland (PSG), a secretion of core-layer component was observed. The PSG lumen exhibits homogeneous staining with azure B under light microscopy (LM) (Fig. 2, top). TEM observation of boxed area (a) of Figure 2 shows that electron-dense secretory globules (core-layer components) are secreted in hemolymph-filled lumen (Fig. 2a). The lumen was filled with core-layer components by accumulation of secretory globules in the middle part of PSG (Fig. 2b). The apical cell had well-developed rough endoplasmic reticulum (RER) and microvilli, suggesting active silk synthesis and secretion.

**Figure 3:**
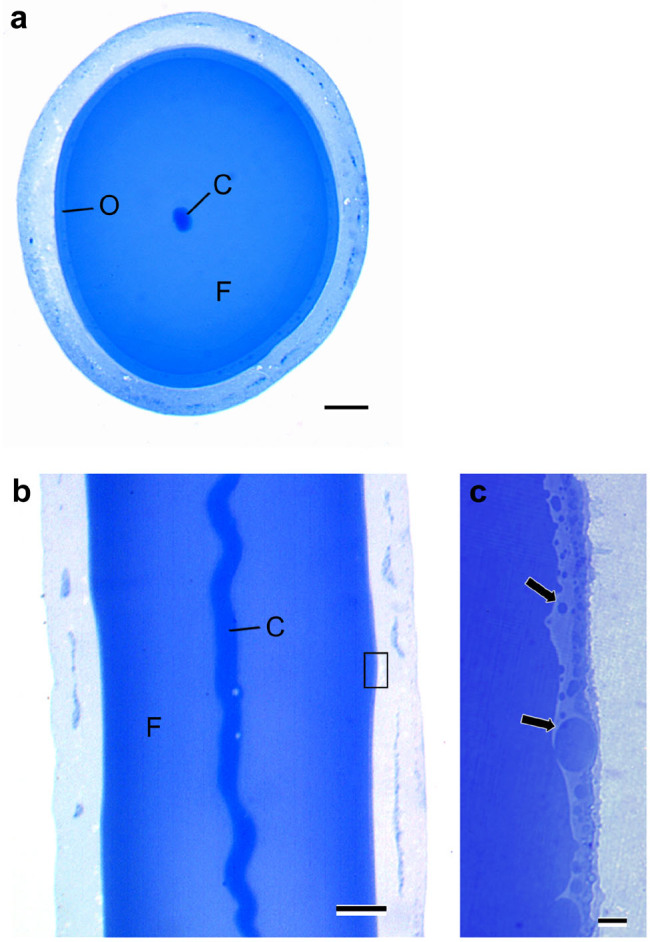
Light micrograph of the middle silk gland. (a) Transverse section of the silk gland. Core layer (C) stained strongly with azure B is located in the center of the *S. marmorata* fibroin layer (F). The outer layer (O), stained faintly, is situated adjacent to the apical cell. Scale bar, 50 μm. (b) Longitudinal section of the silk gland. The core layer (C) is observed as a column shape in the center of the lumen. Scale bar, 50 μm. (c) Enlargement of the boxed area in left micrograph. A large number of *S. marmorata* fibroin secretory globules (arrow) pass through the outer layer and accumulate in the middle silk gland lumen. Scale bar, 10 μm.

The secretion of core-layer components was observed exclusively in a very small area of 1000 μm or less from the posteriormost part of the silk gland. Core-layer components were assembled in the center of the liquid silk in the process of transport toward the spinneret orifice (Fig. 3a,b). As a consequence of the assembly, the core-layer components form column shapes in the hardened silk.

### Middle silk gland

**Figure 4:**
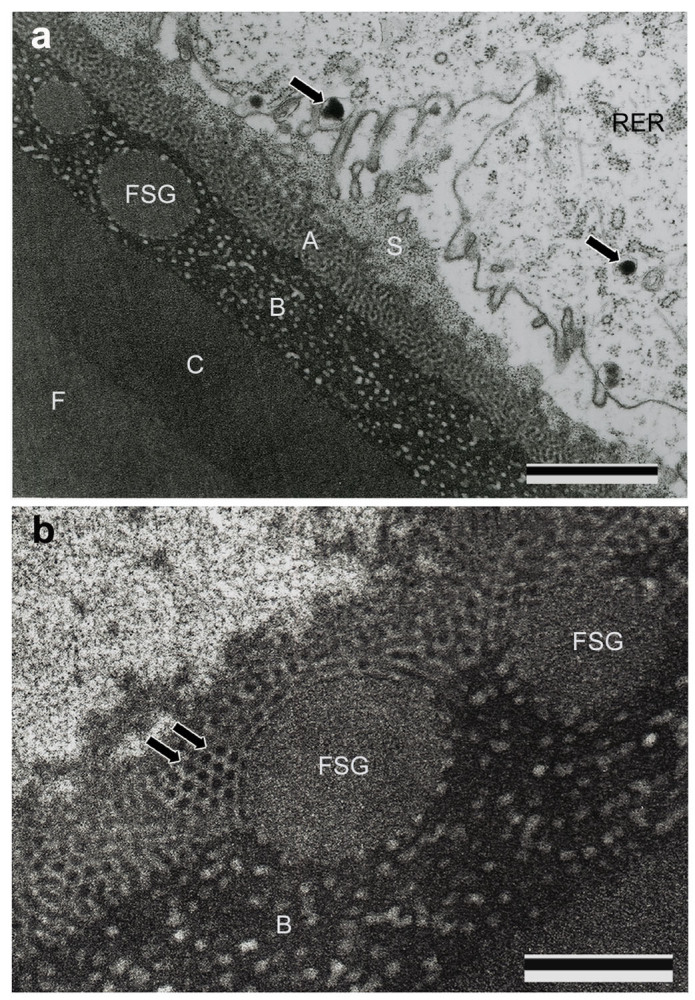
Electron micrograph of the middle silk gland lumen. (a) The outer layer of Figures 3 is separable into three layers (A–C) by TEM observations. A layer: composed of a large number of electron-dense globules. B layer: electron-lucent elliptical materials are present in an electron-dense matrix. C layer: located adjacent to the *S. marmorata* fibroin layer (F) and has no internal structure. Numerous fine granules (S) and electron-dense globules (arrow) are seen around the apical cell, but it is not clear to which layer of the liquid silk these secretions correspond to. Scale bar, 1 μm. RER: rough endoplasmic reticulum. (b) High magnification of the A layer of the middle silk gland lumen. The electron-dense globules are approximately 25 nm in diameter and have almost uniform size and shape (arrow). Scale bar, 500 nm. B: B layer; FSG: *S. marmorata* fibroin secretory globules.

The middle silk gland (MSG) secretes mainly *S. marmorata* fibroin (Sm fibroin), which is the main component of the silk fiber. The liquid silk in MSG lumen was divided into three regions under LM observation: Sm fibroin, core layer, and outer layer (Fig. 3a,b). Several secretory globes passing through the outer layer were seen in the lumen of the marginal zone of the silk gland cells (Fig. 3c). The outer layer of the liquid silk was further distinguished by TEM observation into three different layers: A–C (Fig. 4a). The A layer contained numerous electron-dense globules and was located adjacent to the silk gland cells. The B layer included electron-lucent ellipsoid materials in an electron-dense matrix. The C layer, which was situated adjacent to the Sm fibroin layer, had no internal structure. The electron-dense globule of the A layer had a diameter of approximately 25 nm and an almost perfect spherical shape (Fig. 4b). A large number of secretory granules and electron-dense globules were observed around the boundary between the lumen and the silk gland tissue, but it was unclear as to which components form which layer of the liquid silk (Fig. 4a). Engster (1967) suggested that *P. guttifer* and *N. concinnus* secreted fibroin and a sericin-like material almost entirely in the silk gland. In contrast to those of *B. mori*, Trichoptera silk glands were not clearly divided according to types of silk secretion^18^. Although there is an example of multiple adhesive proteins produced from single-cell glands in barnacles^19^, it was unclear whether multiple proteins are secreted from the same cell in *S. marmorata* MSG.

### Bulbous region

**Figure 5:**
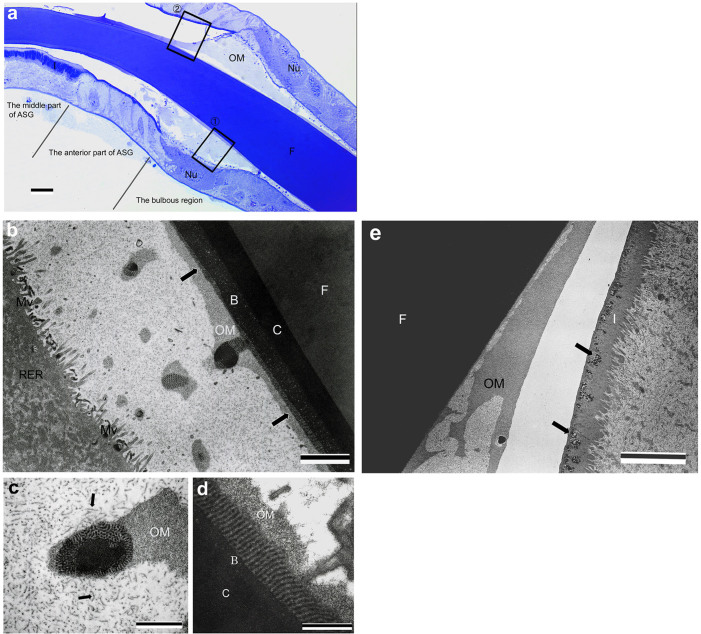
The bulbous region and the anterior part of the anterior silk gland. (a) Light micrograph of the bulbous region and the anterior silk gland in longitudinal section. Liquid silk transported from the middle silk gland is coated with outermost (OM) layer components. Scale bar, 20 μm. F: *S. marmorata* fibroin layer; Nu: nucleus; I: intima. (b–d) Electron micrograph of the bulbous region in longitudinal section (boxed area 1 in Figure 5a). (b) Numerous nanopillar structures (arrow) are seen on the B layer (B) covered with the outermost (OM) layer. The electron-dense globules of the A layer cannot be observed. Scale bar, 2 μm. C: C layer; F: *S. marmorata* fibroin layer; RER: rough endoplasmic reticulum; Mv: microvilli. (c) The nanopillar structures are located on the separated liquid silk (probably the B and C layers), which has accumulated the outermost (OM)-layer components as well. Note that the OM-layer components are observed as a fine fibril (arrow). Scale bar, 1 μm. (d) The nanopillar structure has several round segments that look like a string of beads, which appears to be composed of the globules contained in the A layer of the middle silk gland lumen. Scale bar, 500 nm. B: B layer; C: C layer; OM: outermost layer. (e) Electron micrograph of the anterior part of the anterior silk gland (boxed area 2 in Figure 5a). The intima (I) is composed of bundled microvilli-like structures and has many vesicles that include electron-dense granules in the apical surface (arrow). Scale bar, 5 μm. F: *S. marmorata* fibroin layer; OM: outermost layer.

The bulbous region was located between MSG and the anterior silk gland (ASG), in which the component that forms outermost layer (OM layer) of silk is secreted. The liquid silk transported from MSG was coated with OM-layer components in this region (Fig. 5a). TEM observation of boxed area 1 in Figure 5a revealed that the OM layer is composed of numerous microfibrils approximately 100 nm in length (Fig. 5b). Well-developed RER and microvilli were seen in the apical cells, indicating high metabolic activity. The many pillar structures in a row on the B layer, coated with the OM-layer components, were noteworthy. The pillar structure was hundreds of nanometers long, as was also observed on separated globules of the liquid silk (Fig. 5b,c). The pillar structure had several round-shaped segments, which appeared to have been constructed by electron-dense globules that were included in the A layer of MSG by connection with one another (Fig. 5d). The liquid silk coated with the OM-layer components was transported to ASG, leading to the spinneret.

### Anterior silk gland

In *S. marmorata* and different species of Trichoptera, structural change in the liquid silk that gives rise to an optical anisotropy has been seen in the boundary of the bulbous region and the anterior part of ASG^2^,^18^,^20^. In the anterior part of ASG adjacent to the bulbous region (TEM observation of boxed area 2 in Figure 5a), the intima had microvilli-like structures, the thickness was clearly less than those in the posterior and middle part of ASG, and many vesicles that contain electron-dense granules were located in the epithelial cells (Fig. 5e). The liquid silk coated with the OM layer was spaced apart from the silk gland, and a gap was present between the liquid silk and the intima (Fig. 5e).

**Figure 6:**
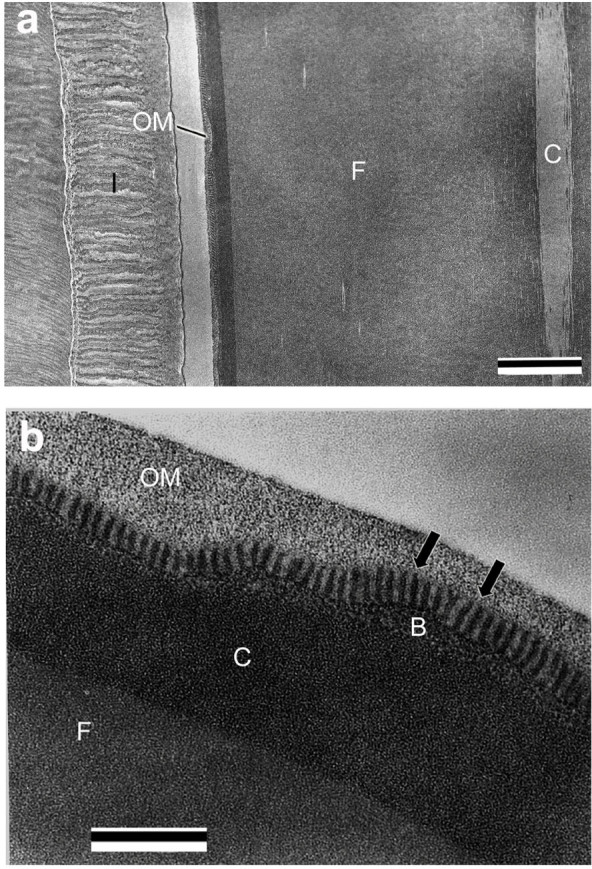
Electron micrograph of the middle part of the anterior silk gland. (a) Longitudinal section of the silk gland. The liquid silk just before spinning underwater is seen in the lumen. In the anterior silk gland, which functions as a duct, the inner surface is covered with a thick cuticular intima (I). Scale bar, 1 μm. C: core layer; F: *S. marmorata* fibroin layer; OM: outermost layer. (b) Transverse section of the liquid silk. The nanopillar structures located at regular intervals (arrow) were observed on the B layer (B), which is coated by the outermost (OM) layer. The average diameter and height of the nanopillar structures are approximately 40 nm and 125 nm, respectively. Scale bar, 500 nm. C: C layer; F: *S. marmorata* fibroin layer.

In the middle and posterior part of ASG, the cuticular intima increased in thickness relative to the anterior part (Fig. 6a). In the lumen, numerous pillar structures (nanopillar structures) covered with the OM layer were observed on the B layer (Fig. 6a,b). Nanopillar structures were approximately 40 nm in width and 125 nm in length and were located at approximately equal spacing on the B layer (Fig. 6b). The contents of B layer changed from electron-lucent ellipsoid materials to electron-dense globules. The thicknesses of the B and C layers were conspicuously reduced in comparison to the liquid silk of MSG and the lumen of the bulbous region. The structural changes appear to be caused by dehydration that occurs when the liquid silk passes from the bulbous region to the anterior part of ASG.

### Hardened silk

**Figure 7:**
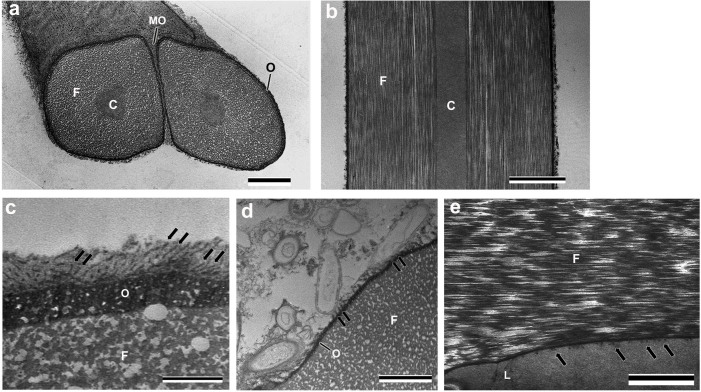
Electron micrograph of hardened silk. (a) Oblique section of the hardened silk. The silk consists of a pair of silk fibers 6–8 μm in width with an oval cross section, which can be divided into four layers: core layer (C) with a shape of column in the center of the fiber, *S. marmorata* fibroin layer (F) composed of numerous fibrils, outer layer (O) containing minute spherical materials, and the outermost (OM) layer gluing the pair of fibers. Scale bar, 2 μm. (b) Longitudinal section of the hardened silk. Numerous fibrils are seen in *S. marmorata* fibroin layer (F). Scale bar, 2 μm. C: core layer. (c) Transverse section of the outermost layer. The outermost layer components adhere to nanopillar structures (arrow). Scale bar, 500 nm. F: *S. marmorata* fibroin layer; O: outer layer. (d) Transverse section of the silk used as capture net. In the surface of capture net silk, usually the outermost layer is peeled off by a water stream, and the nanopillar structures are exposed on the surface. Microorganisms as feed for the larvae are captured on the nanopillar structures (arrow). Scale bar, 1 μm. F: *S. marmorata* fibroin layer; O: outer layer. (e) Cross section of the adhesion layer between the silk and a substrate (leaf). The silk adhesive region is observed as a nonstructural monolayer. The nanopillar structures appear to enter into fine surface roughness of the substrate (arrow). Scale bar, 1 μm. F: *S. marmorata* fibroin layer; L: a leaf.

The hardened silk spun from spinneret consists of a pair of fibers 6–8 μm in width with an oval cross section, which can be distinguished into four layers (Fig. 7a,b). Core-layer shapes a column in the center of the fiber. Sm fibroin layer is the major component of the fiber, which is composed of numerous fibrils. Outer layer contains minute spherical materials, which corresponds to the B and C layers of the liquid silk in the silk gland. The OM layer is exposed on the silk surface, which glues the pair of fibers. In high magnification observation of the silk surface, the nanopillar structures were seen in the OM layer, and numerous OM-layer components adhered to the nanopillar structures (Fig. 7c). In the silk used as capture net, the OM layer is usually removed by a water stream, and the nanopillar structures are exposed on the surface (Fig. 7d). The nanopillar structures were observed capturing microorganisms on the surface (Fig. 7d). In adhesion layer between the silk and a substrate (leaf), the silk adhesive regions (outer layer and OM layer) were observed as a single layer structure, and adhered to the substrate without any gap (Fig. 7e). The nanopillar structures appear to plunge into surface roughness of the substrate (Fig. 7e).

## Discussion

Trichoptera silk fiber formation processes have attracted attention from the viewpoint of studying the differences in environmental adaptations between terrestrial Lepidoptera (butterflies and moths) silk and aquatic silk as well as its adhesive processes. Stewart's group suggests a structural model of Trichoptera silk in which phosphorylated serine repeats (pSX)_4_ of H fibroins complex with the divalent cations Ca^2+^ and Mg^2+^ to form rigid nanocrystalline β-sheet structures^20^,^21^. This fiber formation model would be highly dependent on the local pH and Ca^2+^ concentration within the lumen of the silk gland^3^,^22^. The phosphoserine-rich motifs of Trichoptera silk show similarity to the serine-rich glue protein Pc3 of the marine sandcastle worm (*Phragmatopoma californica*), in that both primary structures have phosphoserine-rich motifs alternating with arginine-rich motifs^23^. The initial solidification model for sandcastle worm glue has been suggested to show complex formation between phosphorylated Pc3 and divalent Ca^2+^/Mg^2+^ triggered by a pH transition from secretory granules (approximately pH 5) to seawater (pH 8.2)^24^. Experiments on *Rhyacophila* (Trichoptera) suggest the possibility that modulation of pH and Ca^2+^ within the lumen of the bulbous region could cause an insolubilization of the liquid silk, which combined with shearing forces gives rise to the observed optical anisotropy^3^. In this study, morphological changes of the liquid silk and histological characteristics of the silk gland cell that suggest pH modulation in the lumen were observed in the boundary of the bulbous region and the anterior part of ASG (Fig. 5).

An H^+^-translocating vacuolar-type ATPase (V-ATPase) has been identified in silk gland cells of *B. mori* and *Samia cynthia ricini* (Eri silkworm) and modulates the pH of the liquid silk, controlling its physicochemical properties^25^,^26^. The liquid silk of *B. mori* changes in rheological properties depending on pH, with a sol–gel transition point estimated at pH 5–6^27^. The pH transition in the middle silk gland of *B. mori* occurs, depending on the growth stage, by a switch of V-ATPase from on to off, resulting in a change from pH 5–6 in vigorously feeding larvae to pH 7–8 in spinning larvae^26^. MSG of *B. mori* functions as a storage tank of liquid fibroin, and neutralization of the spinning larvae MSG is suggested to increase the liquidity of the liquid silk for smooth silk spinning. In ASG of both *B. mori* and *Samia*, V-ATPase is detected in the apical plasma membrane in vigorously feeding larvae, but becomes undetectable in spinning larvae, suggesting that the ASG lumen is neutralized after the onset of spinning^26^,^27^. The pH modulation in the spider major ampullate duct, which secretes dragline silk, is one of the most important factors in the change in rheological properties caused by the action of V-ATPase, as in *B. mori* silk^28^. Microscopic study of the spider *Araneus diadematus* suggests that the distal part of the spinneret of the major ampullate gland uses V-ATPase to transport extracellular H^+^ into the vesicles of the apical cytoplasm and that these H^+^ are used for acidification of the lumen by secretion via exocytosis^29^.

The Trichoptera silk gland probably also has a pH regulation function using V-ATPase; the *S. marmorata* silk gland shows suggestive morphological similarities to the *A. diadematus* spinneret. In the anterior part of ASG, numerous closely packed microvilli that cover the apical surface and many vesicles that include electron-dense granules were observed (Fig. 5e) and are similar to morphological characteristics of the distal part of the *A. diadematus* spinneret. However, the *S. marmorata* ASG differs from *A. diadematus* spinneret in that the vesicles are located in the microvillus intima. In addition, the liquid silk, displaying optical anisotropy, is located apart from the silk gland intima in the anterior part of the ASG lumen (Fig. 5e). The structural change of the liquid silk can be interpreted as insolubilization that leads to fibrillation if pH in the lumen is neutralized due to the transport of H^+^ from the lumen into the apical vesicles by V-ATPase. *S. marmorata* spins silk beginning with the first instar larvae, whereas Lepidoptera spins silk only just before pupation. The increase in luminal pH by transport of H^+^ into the apical vesicles via V-ATPase is possibly a pH modulation adapted for *S. marmorata* larvae that spin silk when necessary.

The distinguishing characteristic of *S. marmorata* silk is the fiber part that is composed of a two-layer structure: a core layer and Sm fibroin (Fig. 7a,b). To our knowledge, in Lepidoptera, closely related to Trichoptera, the silk fibroin is composed of one type. The two layer structure of the fiber part is possibly a feature of capture-net-spinning Trichoptera silk. In TEM observation of casemaker caddisfly silks (*P. guttifer*, *N. concinnus* and Hesperophylax *occidentalis*), the fiber region is composed of a single layer consisting of numerous fine filaments^6^,^30^. In the fiber region of *H. occidentalis* silk, the structural protein having sequence similarity to the PEVK region of the muscle protein has been identified, which is suggested to contribute to the elastic network that provides a restoring force to the self -recovering silk fibers strained past their yield point^6^. The core layer probably has low elasticity and rigidity higher than Sm fibroin composed of fine filaments, and provides silk fiber with the mechanical strength to withstand the forces of rapid currents, allowing the maintenance of a capture net.

The presence of surface-exposed phosphates derived from phosphorylated serine has been confirmed in Trichoptera silk, suggesting the function of promoting adhesion underwater^31^. In thin sections of the silk gland of *Hesperophylax consimilis* (Limnephilidae) and *Arctopsyche grandis* (Hydropsychidae), silk fibers in the anterior silk gland were strongly labeled by phosphorylated serine (pSer) antibody, whereas silk fibers in the middle storage region were poorly labeled^3^. In *S. marmorata*, the OM layer is exposed on the silk surface, which is secreted in the bulbous region located between MSG and ASG. In the anterior silk glands of *H. consimilis* and *A. grandis*, the liquid silk corresponding to the OM layer of *S. marmorata* silk is possibly strongly labeled by pSer antibody. These results suggest that the bulbous region is an important part of the Trichoptera silk gland that secretes an adhesive component containing pSer that contributes to underwater adhesion.

The Smsp-72k protein, containing high numbers of cysteine and charged residues, has been reported to be selectively expressed in the middle and posterior sections of the *S. marmorata* silk gland (probably corresponding to MSG in this study)^32^. The authors suggested that Smsp-72k functions as an underwater adhesive protein and may form intermolecular cross-links between Smsp-72k and fibroin molecules. The *S. marmorata* silk adhesion layer was observed as a single layer in TEM cross section, which is composed of a mixture of the B, C, OM layers, and the nanopillar structures (Fig. 7e). The adhesive layers of *S. marmorata* liquid silk are eventually mixed and adjacent to both Sm fibroin and a substrate. Assuming that Smsp-72k is contained in any one of the A, B, or C layers of MSG lumen, Smsp-72k may play a role in both underwater adhesion and intermolecular linkage to Sm fibroin. In addition, the adhesive region of caddisfly silk is suggested to be dityrosine crosslinked by a peroxidase component of the harden silk^6^. The dityrosine crosslinks also may contribute to the maintenance of a caddisfly silk adhesion by strengthening the linkage of between each layer in the adhesive region.

The most distinguishing characteristic of *S. marmorata* silk is the nanopillar structures in adhesive region (Fig. 6, 7). The role of the nanopillar structures is inferred to increase a contact area of the liquid silk adhesive region and the capture nets from TEM observation. When the silk functions as water adhesive, the nanopillar structures probably works as a brush that is used to apply the liquid silk, and enhance adhesion between the silk and substrates by plunging into nanoscale surface roughness of substrates. The microfibril structure of the OM-layer components may also confer an advantage in underwater adhesion by linking the silk and substrates. When the silk functions as capture nets, the exposed nanopillar structures probably improve the collection efficiency of microorganisms as feeds for the larvae by increasing surface area. In the silk adhesion layer, most of the adhesive region was observed as a nonstructural monolayer (Fig. 7e). The nanopillar structures may be destined to lose their shape in the process of hardening after playing role of plunging into surface roughness of substrates in the initial stage of *S. marmorata* silk adhesion. The nanopillar structures in the silk adhesion layer may be the adhesive region mixture (the B, C, OM layers and nanopillar structures) fitting into surface roughness of substrates.

P. Messersmith's group has shown that gecko-mimetic nanoscale polymer pillars (600 nm in height and 400 nm in diameter) increased wet adhesion nearly 15-fold over uncoated pillar arrays when coated with mussel adhesive protein-mimetic polymer film^33^. Design of *S. marmorata* silk adhesive is similar to gecko/mussel mimetic adhesive in the idea of increasing a contact area of the adhesive face by covering nanostructures with adhesive material.

**Figure 8:**
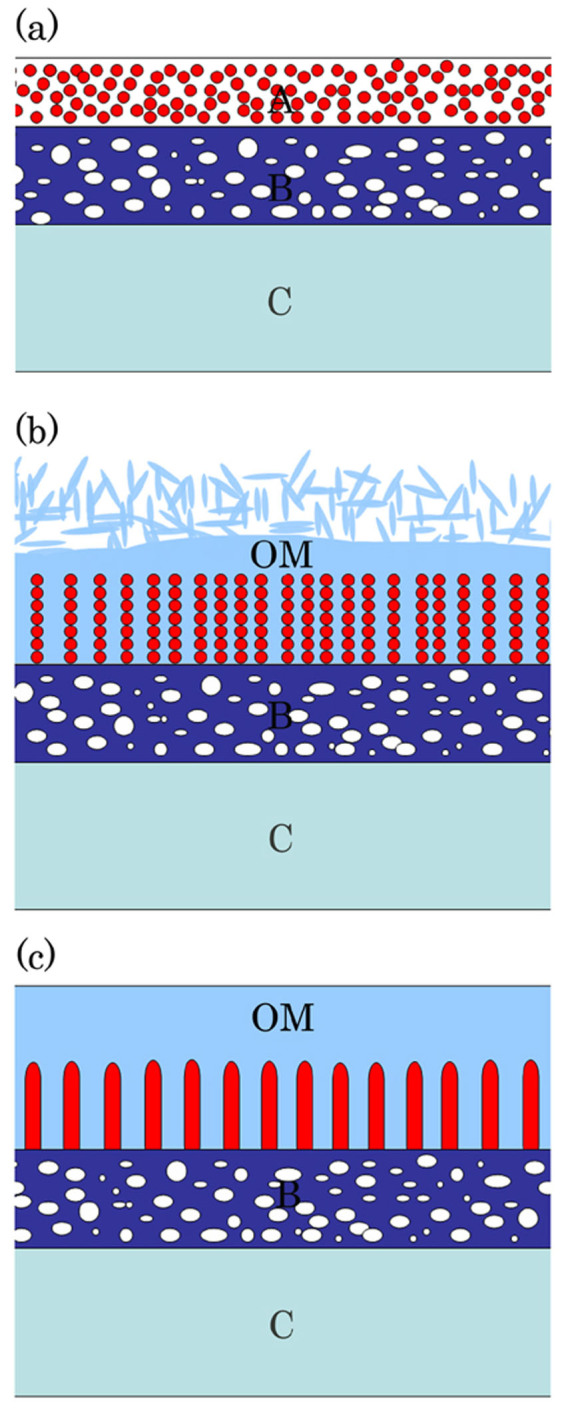
Schematic representation of the nanopillar formation process. (a) The nanopillar precursor materials are present as a large number of electron-dense globules in the A layer of the middle silk gland lumen. (b) Several globules connect to each other to form nanopillar structures on the B layer when the liquid silk is transported to the lumen of the bulbous region. (c) In the anterior silk gland, nanopillars of almost uniform size and shape are situated at regular intervals on the B layer, which is coated with the outermost layer. A: A layer; B: B layer; C: C layer; OM: outermost layer.

The process of nanopillar structure formation is noteworthy, as well as the design of nanostructural adhesive of *S. marmorata* silk (Fig. 8). Although the formation mechanism of the nanopillar structures is unclear, they appear to be formed as a result of environmental changes in the lumen between MSG and the bulbous region.

## Methods

Fifth-instar larvae of *Stenopsyche marmorata* (Fig. 1 insert) were collected from Sagami River. The silk glands were dissected and immediately fixed in a prefixative solution of 2% paraformaldehyde and 2.5% glutaraldehyde for 6 h at room temperature. Divided pieces of the silk glands were repeatedly rinsed with 0.1 M cacodylate buffer and then postfixed in 1.5% OsO_4_ solution for 2 h at room temperature. After dehydration with a graded series of ethanol, the pieces were embedded in Epon 812 (TAAB, Berkshire, UK). For optical microscopic observation, semi-thin sections (0.75 μm) were cut with a glass knife and stained with azure B. Ultrathin sections (70–90 nm) were cut on a Reichert OMU 3 with a 45° diamond knife (Diatome, Biel, Switzerland) and stained with a solution containing lead acetate, lead citrate, and lead nitrate. Stained sections were examined with a Phillips CM120 Biotwin TEM operating at 80 kV accelerating voltage.

## Author Contributions

T.N conceived the research. T.N and T.H. designed the study. T.H. performed the experiments, and wrote the paper.
